# Supplementary material for: TET3 is a regulator and can be targeted for the intervention of myocardial fibrosis
Source: EMBO Mol Med. 2025 Sep 8;17(10):2809–26. doi: 10.1038/s44321-025-00305-4 (PMC12513999; doi:10.1038/s44321-025-00305-4)
Supplement: Supplementary file 1 — Appendix [file 44321_2025_305_MOESM1_ESM.pdf]

Zhu C et al: TET3 is a regulator and can be targeted for the intervention of myocardial fibrosis

Table of Contents for Appendix

Appendix Figure S1…………………………………………..Page 2
Appendix Figure S2…………………………………………..Page 3
Appendix Figure S3…………………………………………..Page 4
Appendix Figure S4…………………………………………..Page 5
Appendix Figure S5…………………………………………..Page 6
Appendix Figure S6…………………………………………..Page 7
Appendix Figure S7…………………………………………..Page 8
Appendix Figure S8…………………………………………..Page 9
Appendix Figure S9…………………………………………..Page 10
Appendix Figure S10…………………………………………..Page 11
Appendix Figure S11…………………………………………..Page 12
Appendix Figure S12…………………………………………..Page 13
Appendix Figure S13…………………………………………..Page 14
Appendix Figure S14…………………………………………..Page 15
Appendix Figure S15…………………………………………..Page 16
Appendix Table S1……………………………………………..Page 17
Appendix Table S2……………………………………………..Page 18
Appendix Table S3……………………………………………..Page 19
Appendix Table S4……………………………………………..Page 20-21

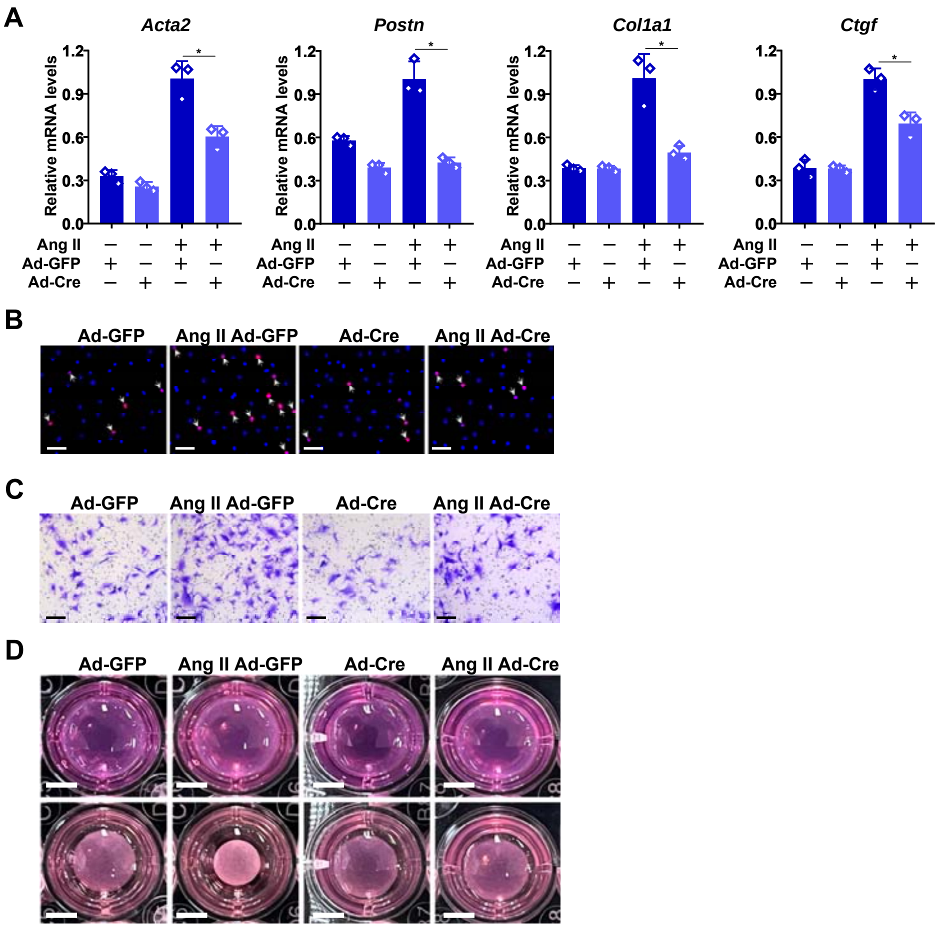

Appendix Appendix Figure S1: (**A-D**) Primary cardiac fibroblasts isolated from Tet3$^{f/f}$ mice were transduced with Ad-Cre or Ad-GFP followed by treatment with Ang II (1μM) for 24h. Myofibroblast markers were examined by qPCR (A). EdU incorporation. Scale bar, 50μm (B). Transwell assay. Scale bar, 50μm (C). Collagen contraction assay. Scale bar, 1cm (D). N=3 biological replicates. Data are expressed as mean±S.D. *, $p < 0.05$, one-way ANOVA with post-hoc Scheff´e.

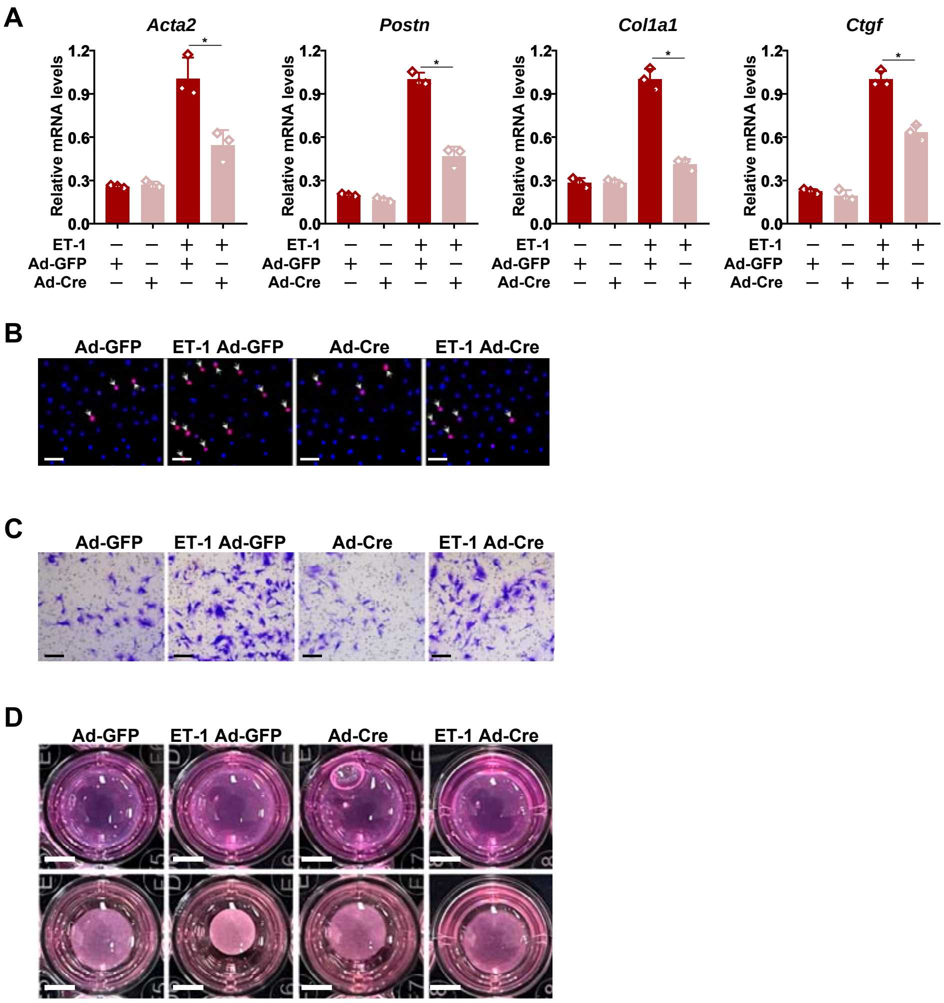

Appendix Figure S2: (**A-D**) Primary cardiac fibroblasts isolated from Tet3$^{f/f}$ mice were transduced with Ad-Cre or Ad-GFP followed by treatment with ET-1 (1μM) for 24h. Myofibroblast markers were examined by qPCR (A). EdU incorporation. Scale bar, 50μm (B). Transwell assay. Scale bar, 50μm (C). Collagen contraction assay. Scale bar, 1cm (D). N=3 biological replicates. Data are expressed as mean±S.D. *, $p<0.05$, one-way ANOVA with post-hoc Scheff´e.

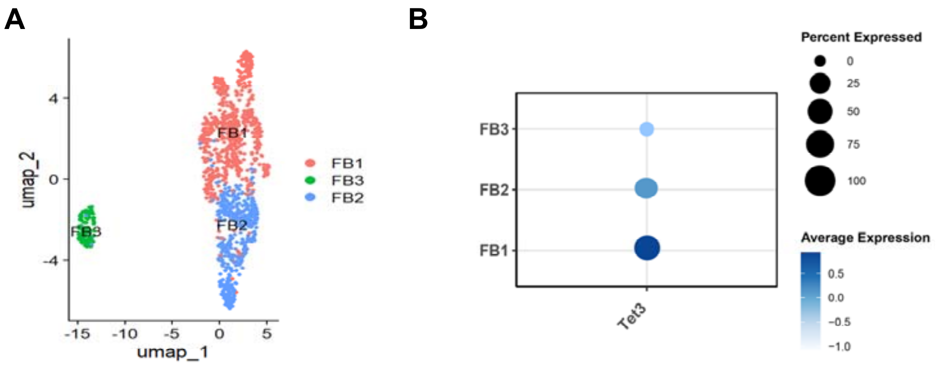

Appendix Figure S3: TET3 expression in different cardiac fibroblast clusters was analyzed using a previously published single-cell RNA-seq dataset (GSE157446). (**A**) UMAP plot of fibroblast subclusters. (**B**) Dot plot showing Tet3-positive cell proportion and mean expression in each fibroblast subclusters.

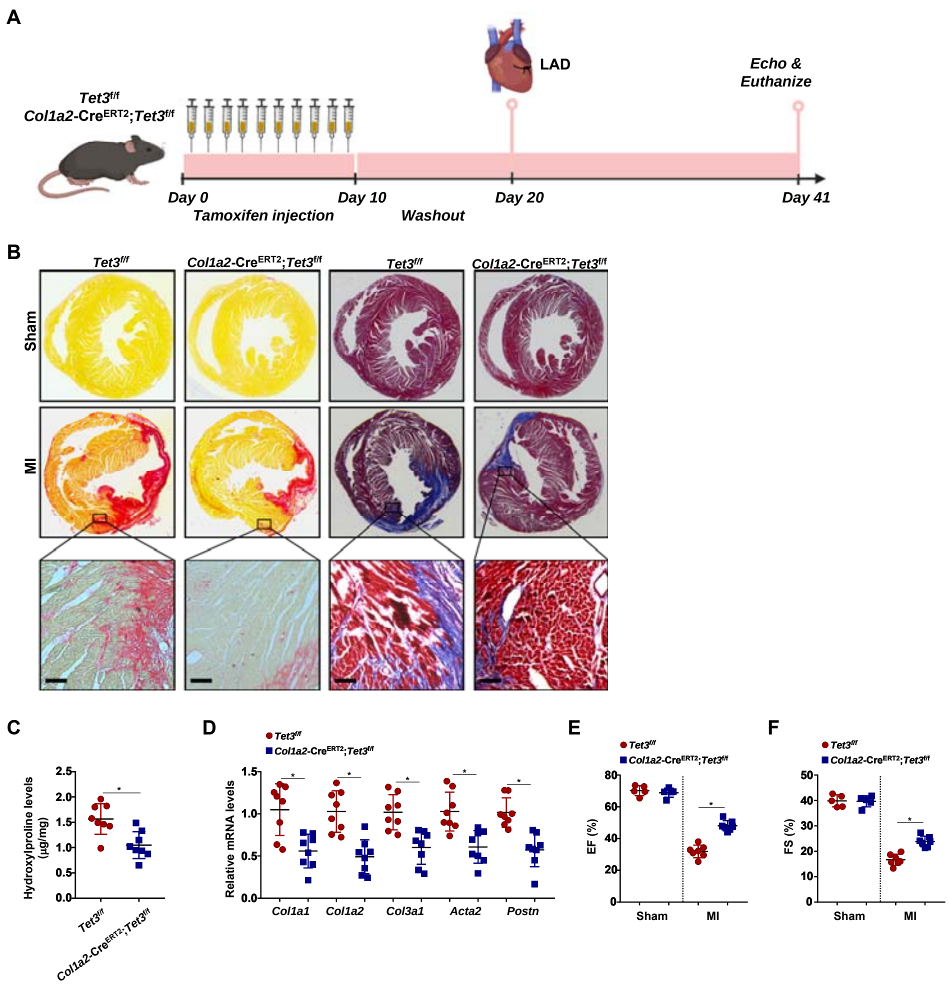

Appendix Figure S4: Fibroblast conditional TET3 knockout and wild type mice were subjected to the LAD procedure and euthanized 3 weeks after the surgery. Scheme of protocol (A). Paraffin sections were stained with PicroSirius Red or Masson's Trichrome (B). Hydroxyproline levels (C). Myofibroblast markers were examined by qPCR (D). LV EF (E) LV FS (F). N=5 mice for the sham groups and N=8 mice for the TAC groups. Scale bar, 50μm. *, $p < 0.05$, one-way ANOVA with post-hoc Scheff′e.

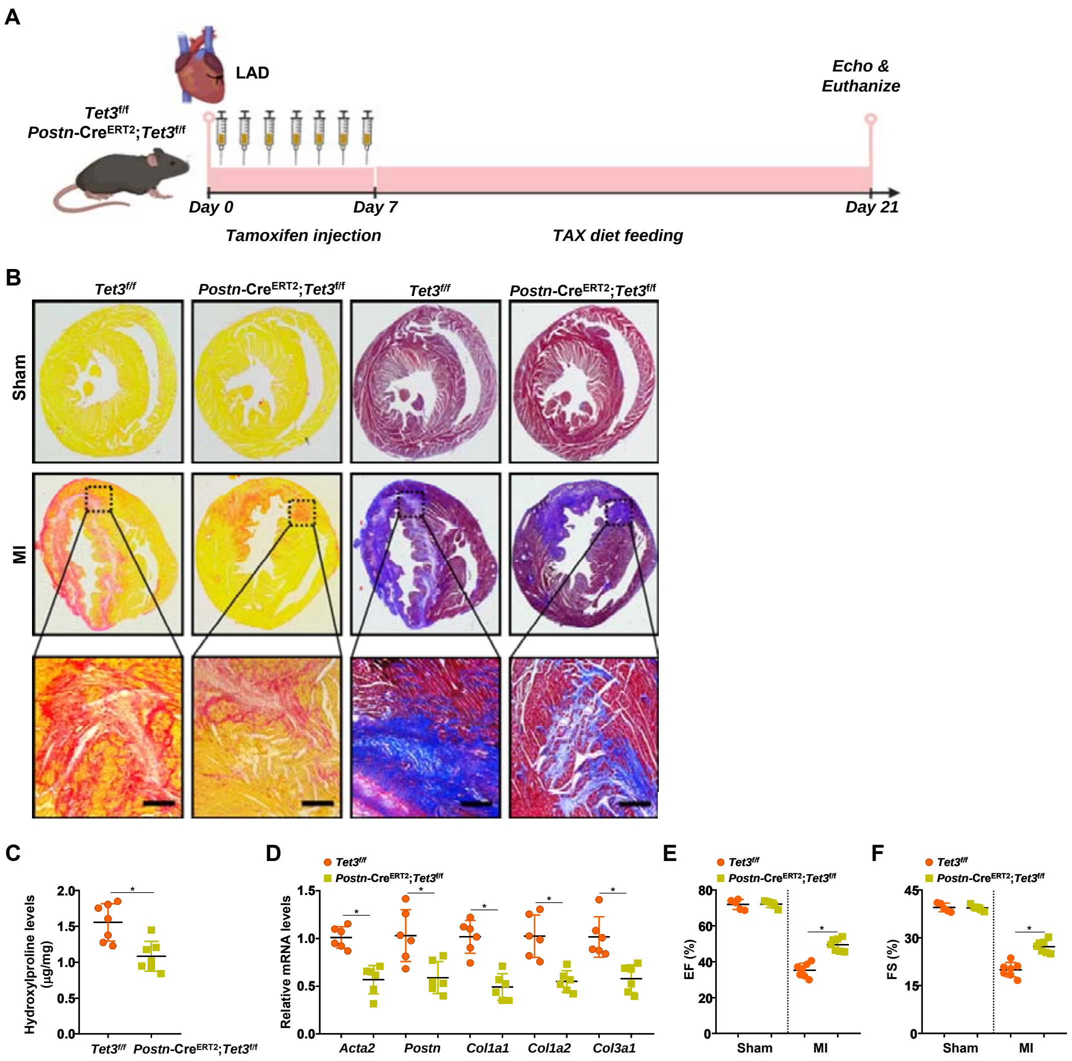

Appendix Figure S5: Myofibroblast conditional TET3 knockout and wild type mice were subjected to the LAD procedure and euthanized 3 weeks after the surgery. Scheme of protocol (A). Paraffin sections were stained with PicroSirius Red or Masson's Trichrome (B). Hydroxyproline levels (C). Myofibroblast markers were examined by qPCR (D). LV EF (E) LV FS (F). N=4 mice for the sham groups and N=7 mice for the TAC groups. Scale bar, 50μm. *, $p < 0.05$, one-way ANOVA with post-hoc Scheff˝e.

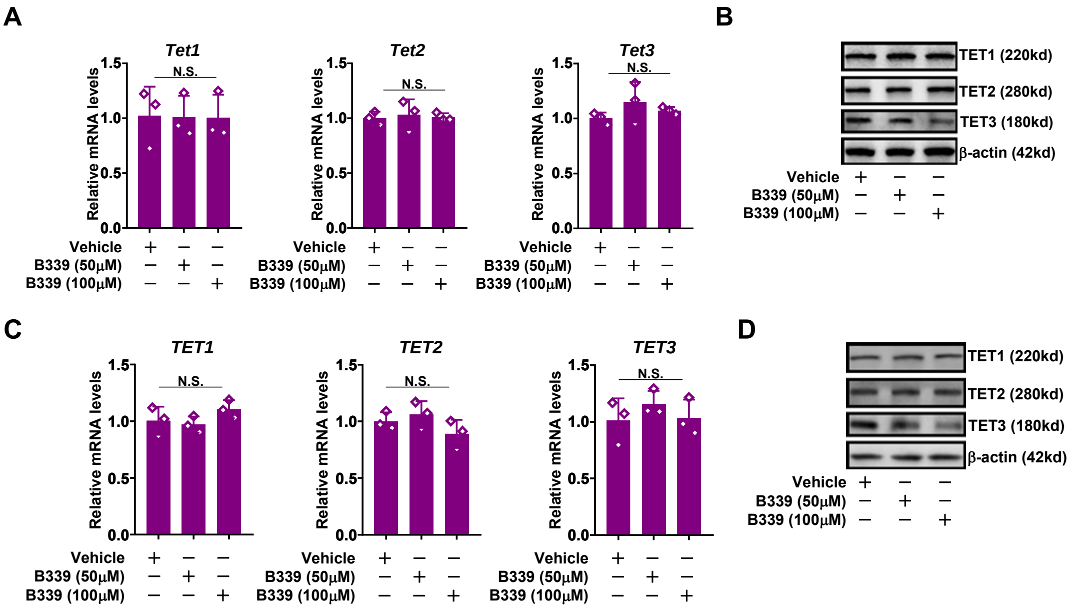

Appendix Figure S6: (**A, B**) Primary murine cardiac fibroblasts were treated with or without Bobcat339 for 24h. TET expression levels were examined by qPCR and Western blotting. N=3 biological replicates. (**C, D**) Primary human cardiac fibroblasts were treated with or without Bobcat339 for 24h. TET expression levels were examined by qPCR and Western blotting. N=3 biological replicates. Data are expressed as mean±S.D. *, $p<0.05$, one-way ANOVA with post-hoc Scheff́e.

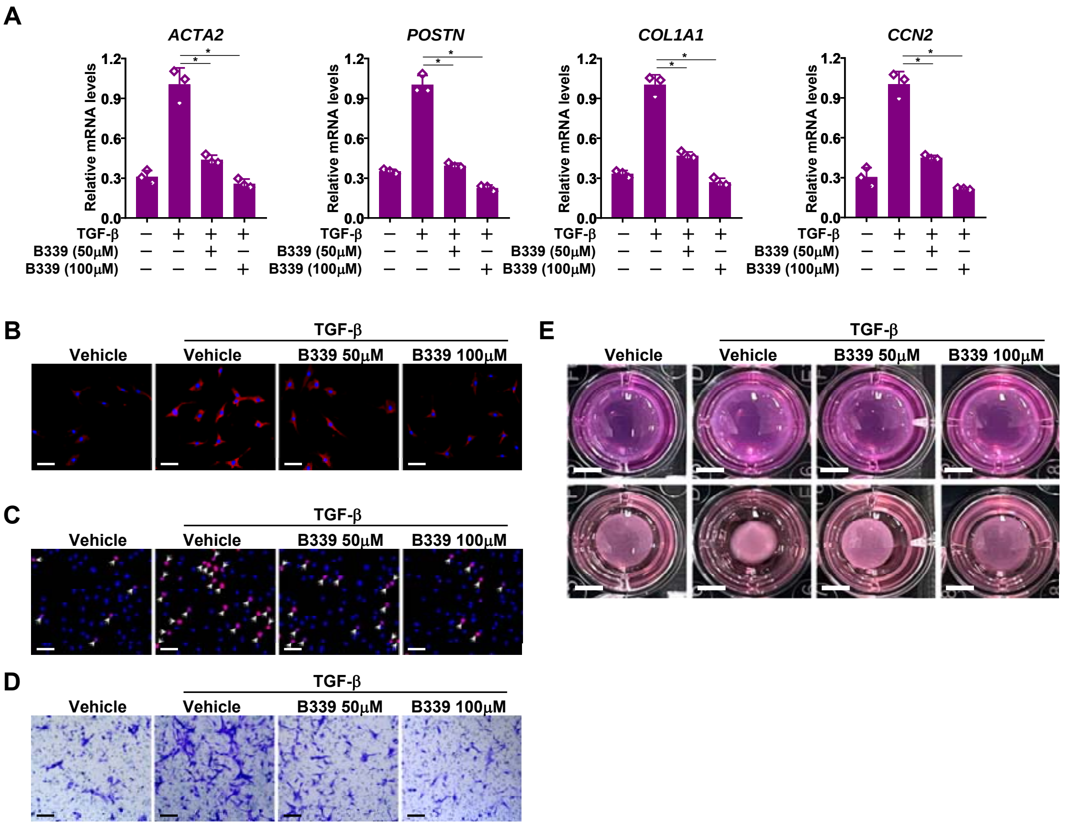

Appendix Figure S7: (**A-E**) Primary human cardiac fibroblasts were treated with TGF-β (5ng/ml) in the presence or absence of Bobcat339 for 24h. Myofibroblast markers were examined by qPCR (A). Immunofluorescence staining with an anti-α-SMA antibody. Scale bar, 50μm (B). EdU incorporation. Scale bar, 50μm (C). Transwell assay. Scale bar, 50μm (D). Collagen contraction assay. Scale bar, 1cm (E). N=3 biological replicates. Data are expressed as mean±S.D. *, $p < 0.05$, one-way ANOVA with post-hoc Scheff′e.

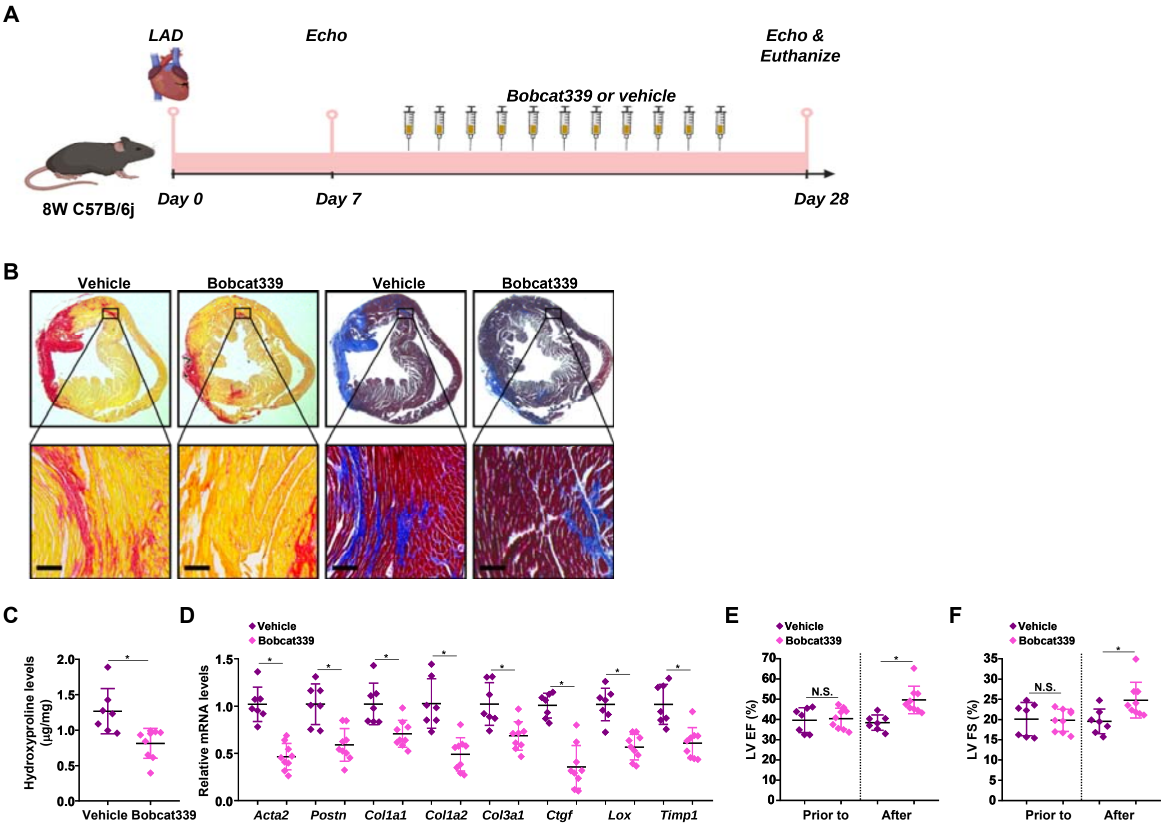

Appendix Figure S8: C57B/6j mice were subjected to the LAD procedure to induce heart failure followed by intervention with Bocat339. Scheme of protocol (A). PicroSirius Red staining and Masson's staining. Scale bar, 50μm (B). Hydroxyproline levels (C). Myofibroblast markers were examined by qPCR (D). Ejection fraction (E). Fractional shortening (F). N=7-9 mice for each group. Data are expressed as mean±S.D. *, $p<0.05$, two-tailed student's test.

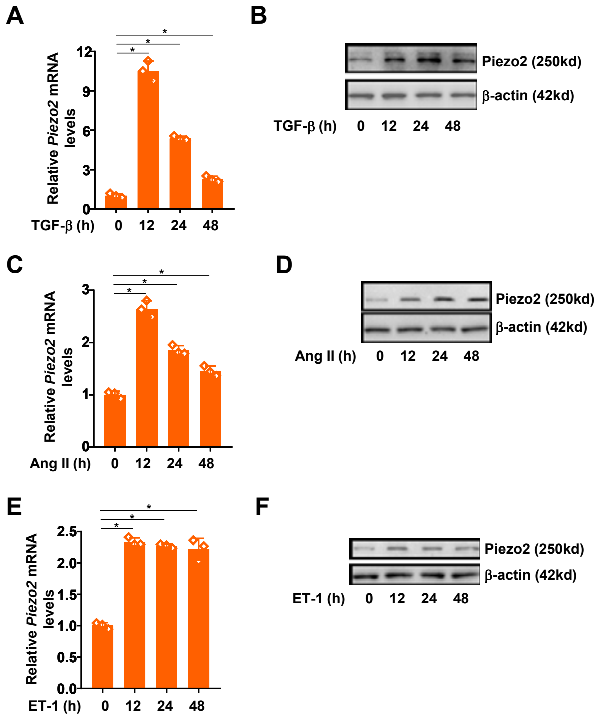

Appendix Figure S9: (**A, B**) Primary murine cardiac fibroblasts were treated with TGF-β (5ng/ml) and harvested at indicated time points. Piezo2 expression was examined by qPCR and Western blotting. (**C, D**) Primary murine cardiac fibroblasts were treated with Ang II (1μM) and harvested at indicated time points. Piezo2 expression was examined by qPCR and Western blotting. (**E, F**) Primary murine cardiac fibroblasts were treated with ET-1 (1μM) and harvested at indicated time points. Piezo2 expression was examined by qPCR and Western blotting. N=3 biological replicates. Data are expressed as mean±S.D. *, $p < 0.05$, one-way ANOVA with post-hoc Scheff´e.

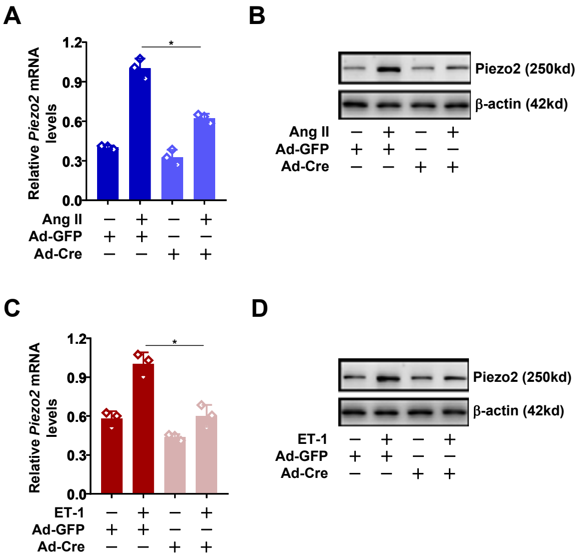

Appendix Figure S10: (**A, B**) Primary cardiac fibroblasts isolated from *Tet3*$^{f/f}$ mice were transduced with Ad-Cre or Ad-GFP followed by treatment with Ang II (1μM) for 24h. Piezo2 expression was examined by qPCR and Western blotting. (**C, D**) Primary cardiac fibroblasts isolated from *Tet3*$^{f/f}$ mice were transduced with Ad-Cre or Ad-GFP followed by treatment with ET-1 (1μM) for 24h. Piezo2 expression was examined by qPCR and Western blotting. N=3 biological replicates. Data are expressed as mean±S.D. *, $p<0.05$, one-way ANOVA with post-hoc Scheff´e.

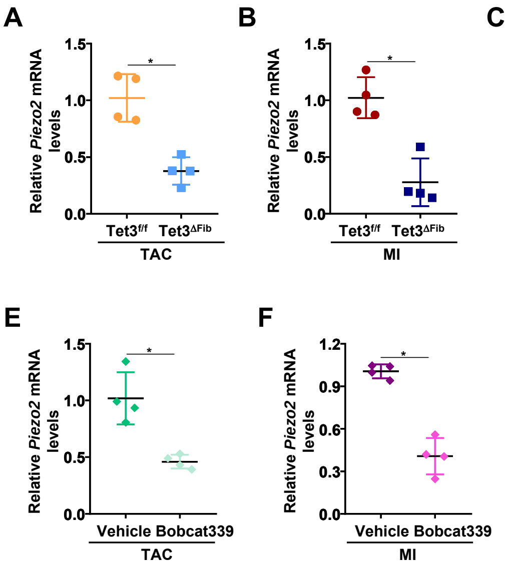

Appendix Figure S11: (**A**) Fibroblast conditional TET3 knockout and wild type mice were subjected to the TAC procedure and euthanized 6 weeks after the surgery. Piezo2 expression was examined by qPCR. (**B**) Fibroblast conditional TET3 knockout and wild type mice were subjected to the LAD procedure and euthanized 3 weeks after the surgery. Piezo2 expression was examined by qPCR. (**C**) Myofibroblast conditional TET3 knockout and wild type mice were subjected to the TAC procedure and euthanized 6 weeks after the surgery. Piezo2 expression was examined by qPCR. (**D**) Myofibroblast conditional TET3 knockout and wild type mice were subjected to the LAD procedure and euthanized 3 weeks after the surgery. Piezo2 expression was examined by qPCR. (**E**) C57B/6j mice were subjected to the TAC procedure to induce heart failure followed by intervention with Bocat339. Piezo2 expression was examined by qPCR. (**F**) C57B/6j mice were subjected to the LAD procedure to induce heart failure followed by intervention with Bocat339.. Piezo2 expression was examined by qPCR. N=4 mice for each group. Data are expressed as mean±S.D. *, $p<0.05$, two-tailed student's test.

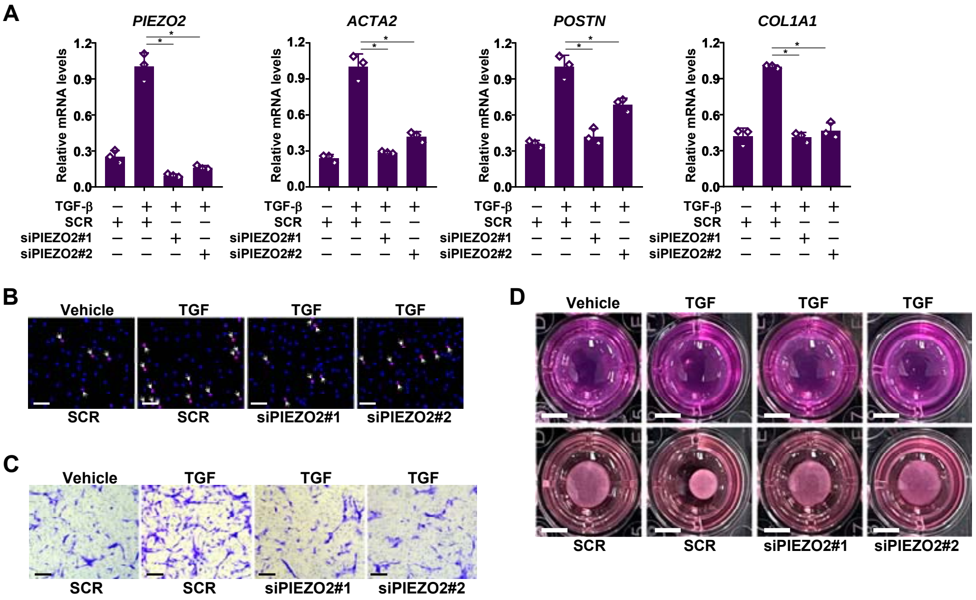

Appendix Figure S12: (A-D) Human primary cardiac fibroblasts were transfected with siRNAs targeting PIEZO2 or scrambled siRNA (SCR) followed by treatment with TGF-β (5ng/ml) for 24h. Myofibroblast markers were examined by qPCR (A). EdU incorporation. Scale bar, 50μm (B). Transwell assay. Scale bar, 50μm (C). Collagen contraction assay. Scale bar, 1cm (D). N=3 biological replicates. Data are expressed as mean±S.D. *, $p < 0.05$, one-way ANOVA with post-hoc Scheff´e.

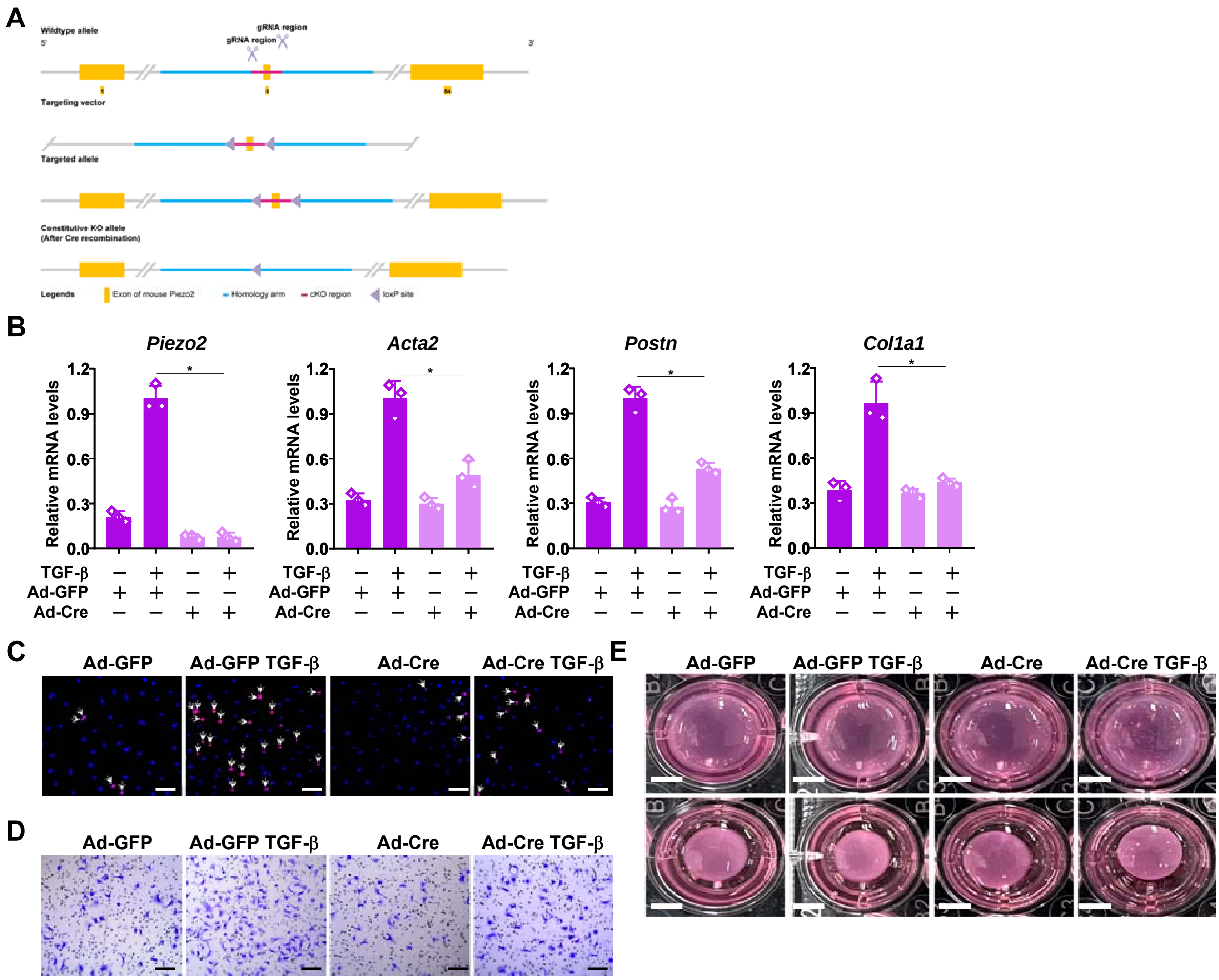

Appendix Figure S13: Primary cardiac fibroblasts isolated from Piezo2$^{f/f}$ mice were transduced with Ad-Cre or Ad-GFP followed by treatment with TGF-β (5ng/ml) for 24h. Targeting scheme (A). Myofibroblast markers were examined by qPCR (B). EdU incorporation. Scale bar, 50μm (C). Transwell assay. Scale bar, 50μm (D). Collagen contraction assay. Scale bar, 1cm (E). N=3 biological replicates. Data are expressed as mean±S.D. *, $p < 0.05$, one-way ANOVA with post-hoc Scheff´e.

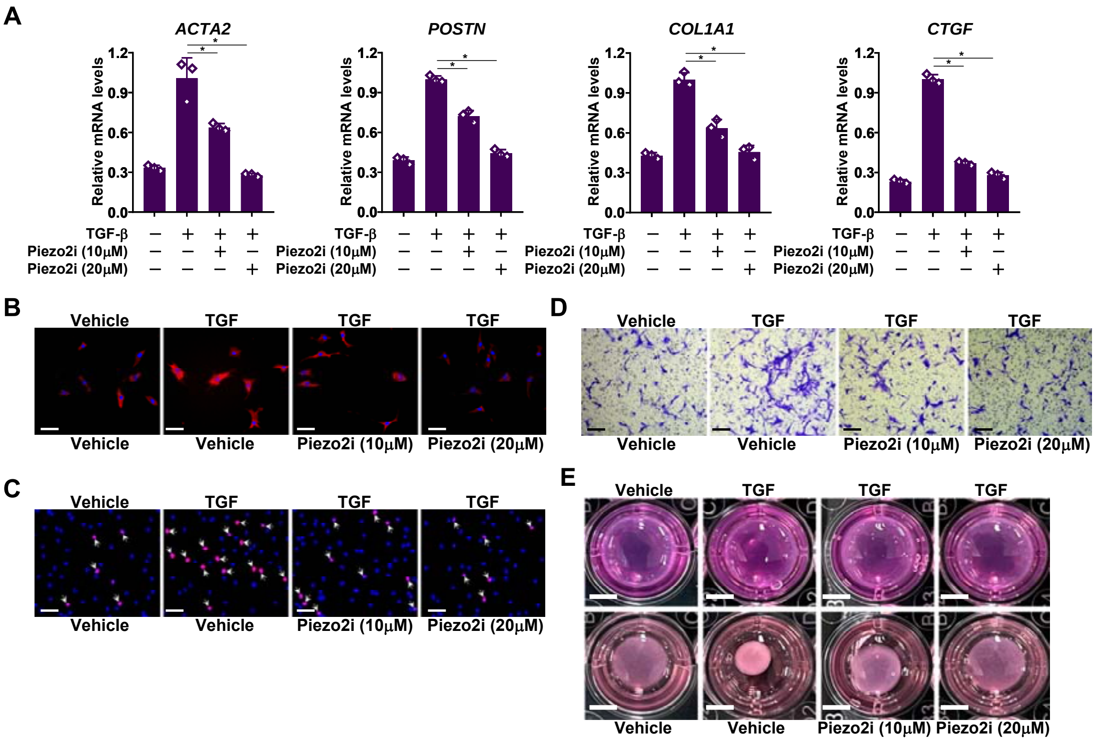

Appendix Figure S14: (**A-E**) Primary human cardiac fibroblasts were treated with TGF-β (5ng/ml) in the presence or absence of Piezo2i for 24h. Myofibroblast markers were examined by qPCR (A). Immunofluorescence staining with an anti-α-SMA antibody. Scale bar, 50μm (B). EdU incorporation. Scale bar, 50μm (C). Transwell assay. Scale bar, 50μm (D). Collagen contraction assay. Scale bar, 1cm (E). N=3 biological replicates. Data are expressed as mean±S.D. *, $p < 0.05$, one-way ANOVA with post-hoc Scheff′e.

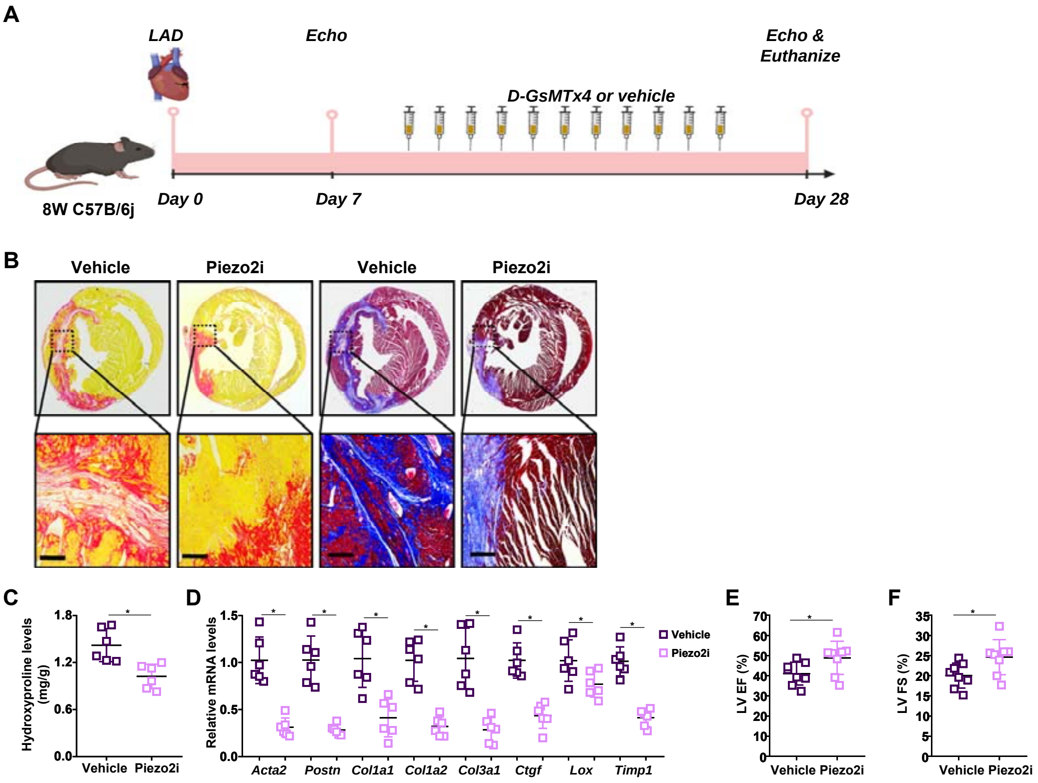

Appendix Figure S15: C57B/6j mice were subjected to the LAD procedure to induce heart failure followed by intervention with Piezo2i. Scheme of protocol (A). PicroSirius Red staining and Masson's staining. Scale bar, 50μm (B). Hydroxyproline levels (C). Myofibroblast markers were examined by qPCR (D). Ejection fraction (E). Fractional shortening (F). N=6-8 mice for each group. Data are expressed as mean±S.D. *, $p<0.05$, two-tailed student's test.

Appendix Table S1: QPCR Primers Sequences

**Gene**	**Forward primer**	**Reverse primer**
Mouse *Col1a1*	GCTCCTCTTAGGGGCCACT	ATTGGGGACCCTTAGGCCAT
Mouse *Col3a1*	CTGTAACATGGAAACTGGGGAAA	CCATAGCTGAACTGAAAACCACC
Mouse *Acta2*	CCCAGACATCAGGGAGTAATGG	TCTATCGGATACTTCAGCGTCA
Mouse *Col1a2*	TCGTGCCTAGCAACATGCC	TTTGTCAGAATACTGAGCAGCAA
Mouse *Ctgf*	GGCCTCTTCTGCGATTTCG	GCAGCTTGACCCTTCTCGG
Mouse *Postn*	CCTGCCCTTATATGCTCTGCT	AAACATGGTCAATAGGCATCACT
Mouse *Lox*	CAGCCACATAGATCGCATGGT	GCCGTATCCAGGTCGGTTC
Mouse *Tet3*	TGCGATTGTGTCGAACAAATAGT	TCCATACCGATCCTCCATGAG
Mouse *Piezo2*	GTGGTATGCAACCCAGTACCC	GGCCATTCTCTATGGGCAGG
Mouse *Timp1*	CGAGACCACCTTATACCAGCG	ATGACTGGGGTGTAGGCGTA
Human *COL1A1*	GAGGGCCAAGACGAAGACATC	CAGATCACGTCATCGCACAAC
Human *COL3A1*	GGAGCTGGCTACTTCTCGC	GGGAACATCCTCCTTCAACAG
Human *ACTA2*	AAAAGACAGCTACGTGGGTGA	GCCATGTTCTATCGGGTACTTC
Human *POSTN*	CTCATAGTCGTATCAGGGGTCG	ACACAGTCGTTTTCTGTCCAC
Human *TET3*	GCCGGTCAATGGTGCTAGAG	CGGTTGAAGGTTTCATAGAGCC
Human *TIMP1*	CTTCTGCAATTCCGACCTCGT	ACGCTGGTATAAGGTGGTCTG
Human *PIEZO2*	ATGGCCTCAGAAGTGGTGTG	ATGTCCTTGCATCGTCGTTTT

Appendix Table S2: Antibody Information

Antigen	Vendor (catalog#)	Application
β-actin	Sigma (A2228)	IB
TET1	Invitrogen (GT1462)	IB
TET2	Abcam (Ab94580)	IB
TET3	Active Motif (61395)	IB, CUT&Tag-seq
SRF	Cell Signaling Tech (5147)	CUT&Tag-seq
TAZ	Cell Signaling Tech (83669)	CUT&Tag-seq
Piezo2	Proteintech (26205-1)	IB
α-SMA	Abcam (ab150301)	IF

Appendix Table S3: Patient information

ID	Gender (M/F)	Age (Yr)	cTnT (ng/L)	Total cholesterol (mM)	LV EF (%)	
1	M	56	19	4.48	25	
2	M	54	270	4.41	36	
3	F	68	16	6.38	31	
4	M	59	71	3.19	11	
5	F	8	22	3.39	40	
6	M	51	29	3.29	37	
7	F	67	47	3.20	26	
8	M	51	48	1.68	26	
9	M	51	51	2.15	39	

Appendix Table S4: Exact p values

Figure #	Statistical method	Groups	Exact *p* value
Figure 1A TET3 mRNA	One way ANOVA	SCR vs siTET3#1	0.0011
	One way ANOVA	SCR vs siTET3#2	0.00041
Figure 1A ACTA2 mRNA	One way ANOVA	SCR vs siTET3#1	0.0033
	One way ANOVA	SCR vs siTET3#2	0.00042
Figure 1A POSTN mRNA	One way ANOVA	SCR vs siTET3#1	0.0085
	One way ANOVA	SCR vs siTET3#2	0.00055
Figure 1A COL1A1 mRNA	One way ANOVA	SCR vs siTET3#1	0.00075
	One way ANOVA	SCR vs siTET3#2	0.00017
Figure 1A CCN2 mRNA	One way ANOVA	SCR vs siTET3#1	6.80E-05
	One way ANOVA	SCR vs siTET3#2	6.40E-06
Figure 1F Acta2 mRNA	One way ANOVA	Ad-GFP TGF vs Ad-Cre TGF	0.00073
Figure 1F Postn mRNA	One way ANOVA	Ad-GFP TGF vs Ad-Cre TGF	0.0025
Figure 1F Ccn2 mRNA	One way ANOVA	Ad-GFP TGF vs Ad-Cre TGF	0.0012
Figure 1F Col1a1 mRNA	One way ANOVA	Ad-GFP TGF vs Ad-Cre TGF	0.00099
Figure 2G Acta2 mRNA	two-tailed student's test	FCKO vs WT	0.00088
Figure 2G Postn mRNA	two-tailed student's test	FCKO vs WT	0.00023
Figure 2G Col1a1 mRNA	two-tailed student's test	FCKO vs WT	0.001
Figure 2G Col1a2 mRNA	two-tailed student's test	FCKO vs WT	0.0022
Figure 2G Col3a1 mRNA	two-tailed student's test	FCKO vs WT	0.002
Figure 2G Ctgf mRNA	two-tailed student's test	FCKO vs WT	0.0064
Figure 2G Lox mRNA	two-tailed student's test	FCKO vs WT	0.00021
Figure 2G Timp1 RNA	two-tailed student's test	FCKO vs WT	0.00049
Figure 2H	two-tailed student's test	FCKO TAC vs WT TAC	0.0037
Figure 2I	two-tailed student's test	FCKO TAC vs WT TAC	0.016
Figure 2J	two-tailed student's test	FCKO TAC vs WT TAC	0.022
Figure 2Q Acta2 mRNA	two-tailed student's test	FCKO vs WT	0.00076
Figure 2Q Postn mRNA	two-tailed student's test	FCKO vs WT	0.00043
Figure 2Q Col1a1 mRNA	two-tailed student's test	FCKO vs WT	8.80E-05
Figure 2Q Col1a2 mRNA	two-tailed student's test	FCKO vs WT	0.00025
Figure 2Q Col3a1 mRNA	two-tailed student's test	FCKO vs WT	0.00046
Figure 2Q Ctgf mRNA	two-tailed student's test	FCKO vs WT	0.00036
Figure 2Q Lox mRNA	two-tailed student's test	FCKO vs WT	0.00078
Figure 2Q Timp1 RNA	two-tailed student's test	FCKO vs WT	0.00017
Figure 2R	two-tailed student's test	FCKO TAC vs WT TAC	0.002
Figure 2S	two-tailed student's test	FCKO TAC vs WT TAC	0.00044
Figure 2T	two-tailed student's test	FCKO TAC vs WT TAC	0.00035
Figure 3A Acta2 mRNA	One way ANOVA	B339 (50) vs TGF	0.0015
	One way ANOVA	B339 (100) TGF	0.00049
Figure 3A Postn mRNA	One way ANOVA	B339 (50) vs TGF	0.014
	One way ANOVA	B339 (100) TGF	0.0015
Figure 3A Col1a1 mRNA	One way ANOVA	B339 (50) vs TGF	0.013
	One way ANOVA	B339 (100) TGF	0.002
Figure 3A Ctgf mRNA	One way ANOVA	B339 (50) vs TGF	0.00049
	One way ANOVA	B339 (100) TGF	0.00039
Figure 3L	two-tailed student's test	B339 vs vehicle	0.008
Figure 3M Acta2 mRNA	two-tailed student's test	B339 vs vehicle	6.40E-07
Figure 3M Postn mRNA	two-tailed student's test	B339 vs vehicle	2.20E-05
Figure 3M Col1a1 mRNA	two-tailed student's test	B339 vs vehicle	0.00047
Figure 3M Col1a2 mRNA	two-tailed student's test	B339 vs vehicle	0.00013
Figure 3M Col3a1 mRNA	two-tailed student's test	B339 vs vehicle	0.0023
Figure 3M Ctgf mRNA	two-tailed student's test	B339 vs vehicle	7.10E-06
Figure 3M Lox mRNA	two-tailed student's test	B339 vs vehicle	0.00086
Figure 3M Timp1 RNA	two-tailed student's test	B339 vs vehicle	0.0003
Figure 3N	two-tailed student's test	B339 vs vehicle	0.0053
Figure 3O	two-tailed student's test	B339 vs vehicle	0.0089

Figure 4M	One way ANOVA	Ad-GFP TGF vs Ad-Cre TGF	0.00044
Figure 4O	One way ANOVA	SCR vs siTET3#1	0.00037
	One way ANOVA	SCR vs siTET3#2	0.00035
Figure 4Q	One way ANOVA	B339 (50) vs TGF	0.0022
	One way ANOVA	B339 (100) TGF	0.00048
Figure 4S	One way ANOVA	B339 (50) vs TGF	0.00053
	One way ANOVA	B339 (100) TGF	3.60E-05
Figure 4U	One way ANOVA	12h vs 0h	0.00064
	One way ANOVA	24h vs 0h	0.0008
	One way ANOVA	48h vs 0h	0.011
Figure 4V	One way ANOVA	Ad-GFP TGF vs Ad-Cre TGF	0.0033
Figure 5A Acta2 mRNA	One way ANOVA	Piezo2i (10) vs TGF	0.0089
	One way ANOVA	Piezo2i (20) vs TGF	0.0018
Figure 5A Postn mRNA	One way ANOVA	Piezo2i (10) vs TGF	0.0056
	One way ANOVA	Piezo2i (20) vs TGF	0.0013
Figure 5A Col1a1 mRNA	One way ANOVA	Piezo2i (10) vs TGF	0.0024
	One way ANOVA	Piezo2i (20) vs TGF	0.00027
Figure 5A Ctgf mRNA	One way ANOVA	Piezo2i (10) vs TGF	0.0012
	One way ANOVA	Piezo2i (20) vs TGF	0.00017
Figure 5L	two-tailed student's test	Piezo2i vs vehicle	0.0065
Figure 5M Acta2 mRNA	two-tailed student's test	Piezo2i vs vehicle	0.00013
Figure 5M Postn mRNA	two-tailed student's test	Piezo2i vs vehicle	4.70E-05
Figure 5M Col1a1 mRNA	two-tailed student's test	Piezo2i vs vehicle	0.00079
Figure 5M Col1a2 mRNA	two-tailed student's test	Piezo2i vs vehicle	0.0067
Figure 5M Col3a1 mRNA	two-tailed student's test	Piezo2i vs vehicle	0.0023
Figure 5M Ctgf mRNA	two-tailed student's test	Piezo2i vs vehicle	0.0003
Figure 5M Lox mRNA	two-tailed student's test	Piezo2i vs vehicle	0.0028
Figure 5M Timp1 RNA	two-tailed student's test	Piezo2i vs vehicle	0.00062
Figure 5N	two-tailed student's test	Piezo2i vs vehicle	0.00081
Figure 5O	two-tailed student's test	Piezo2i vs vehicle	0.00052
